# A comparison between self-report and interviewer-rated retrospective reports of childhood abuse among individuals with first-episode psychosis and population-based controls

**DOI:** 10.1016/j.jpsychires.2020.02.002

**Published:** 2020-04

**Authors:** Charlotte Gayer-Anderson, Ulrich Reininghaus, Isabell Paetzold, Kathryn Hubbard, Stephanie Beards, Valeria Mondelli, Marta Di Forti, Robin M. Murray, Carmine M. Pariante, Paola Dazzan, Thomas J. Craig, Helen L. Fisher, Craig Morgan

**Affiliations:** a Institute of Psychiatry, Psychology & Neuroscience, King's College London, UK; b ESRC Centre for Society and Mental Health, King's College London, UK; c Department of Public Mental Health, Central Institute of Mental Health, Medical Faculty Mannheim, University of Heidelberg, Mannheim, Germany; d National Institute for Health Research (NIHR), Mental Health Biomedical Research Centre at South London, Maudsley NHS Foundation Trust, King's College London, UK

**Keywords:** Case-control study, Maltreatment, Measurement, Psychometric, Psychotic disorder, Validity

## Abstract

The typical reliance on self-report questionnaires in retrospective case-control studies of childhood abuse and psychotic disorders has been criticised, due to the potential for recall bias associated with, amongst other factors, cognitive impairments and detachment from reality, among individuals with psychosis. One way to establish if any substantial bias may exist is to examine whether the concordance of reports of childhood abuse established from retrospective self-report methods versus more comprehensive interviewer-rated assessments differ between individuals with psychosis and controls. Data from the Childhood Adversity and Psychosis (CAPsy) study were used to examine the accuracy, strength of agreement, and convergent validity of two distinct retrospective measures of childhood abuse: a self-report questionnaire (the Childhood Trauma Questionnaire; CTQ) and a comprehensive interview (the Childhood Experiences of Care and Abuse schedule; CECA). In a sample of 234 cases with first-episode psychosis and 293 controls, we found no strong evidence that the validity of the two measures differed between cases and controls. For reports of sexual and emotional abuse, we found fair levels of agreement between CECA and CTQ ratings in both groups (kappa coefficients 0.43–0.53), moderate to high sensitivity and specificity, and reasonably high convergent validity (tetrachoric correlations of 0.78–0.80). For physical abuse, convergent validity was slightly lower in cases compared with controls. Both measures can be used in future studies to retrospectively assess associations between childhood abuse and psychotic phenomena, but time-permitting, the CECA is preferable as it provides additional important contextual details of abuse exposure.

## 1 Introduction

A large body of research has consistently reported that childhood abuse (e.g. physical, sexual or emotional), along with other forms of maltreatment, is associated with an increased risk of psychosis (Matheson et al., 2013; Morgan and Gayer-Anderson, 2016; Varese et al., 2012). Despite this, the validity of the reported association is still questioned, largely because the reliance on retrospective reports of childhood abuse may bias findings (Susser and Widom, 2012).

Bias will occur if the validity of recall varies by outcome status (i.e., presence of psychosis or not). It may be, for example, that recall is less accurate among those with a psychotic disorder because of cognitive impairments (Saykin et al., 1991), depressed mood (Colman et al., 2016), delusional beliefs and detachment from reality (Lysaker et al., 2005), and patients’ attempts to explain their illness (Susser and Widom, 2012). It is not clear, however, whether such processes will lead to under- or over-reporting. Some have suggested that those with mental health problems are liable to over-report childhood adverse experiences (Colman et al., 2016), while others have reported that patients may actually be more likely to under-report abuse histories (Dill et al., 1991; Read et al., 2005). Whilst these methodological issues imply that prospective longitudinal designs are optimal for studying childhood abuse and psychosis (Susser and Widom, 2012), investigators remain largely dependent on retrospective reports of childhood adversity, given that prospective studies are prohibitively expensive for rare outcomes such as psychosis.

In the absence of a gold standard instrument for measuring childhood abuse retrospectively, some have questioned the convergent validity of different measures (Dill et al., 1991; Morgan and Fisher, 2007). It has been argued that investigator-based interviews have greater merits over and above self-report questionnaires of childhood trauma (Bifulco et al., 1997; Roy and Perry, 2004). Such methods, with the use of guided questions to elicit detailed narratives of childhood experiences, have the benefit of being less affected by some reporting biases, and use a more standardised approach to ratings with the inclusion of manualised examples. They also allow for assessment of the complex multi-dimensional nature of childhood abuse, i.e. the age of occurrence, perpetrator, frequency, as well as severity. However, interviews also have their disadvantages: they are lengthy to administer, and reliable implementation and scoring normally requires intensive training. In many cases, a more cost-effective method for acquiring this information is needed. As such, a self-report questionnaire may be an alternative, and are more typically used, in those instances.

It is therefore imperative that we more fully understand, in individuals with psychosis, the degree to which retrospective reports of childhood abuse established from self-report questionnaire-based measures correspond with ratings from interview-administered assessments. Therefore, using data from both patients with first-episode psychosis and population-based controls, we aimed to investigate the: i. accuracy, ii. strength of agreement (as specific types of reliability), and iii. convergent validity of childhood abuse reports obtained using two distinct measures: a self-report questionnaire and an interview-based measure.

## 2 Methods

### 2.1 Participants

The sample for this study was drawn from individuals who participated in the Childhood Adversity and Psychosis (CAPsy) study, a population-based case-control study of first-episode psychosis, conducted between 2010 and 2014. Full details of the study, and participant recruitment, are provided elsewhere (Beards et al., in press). Briefly, cases were aged between 18 and 64 years old, living within defined catchment areas in south-east London, UK, who presented to mental health services for the first time with psychosis (ICD-10 diagnoses of F20-29 and F30-33). Exclusion criteria were: evidence of an organic cause; transient psychotic symptoms resulting from acute intoxication as defined by ICD-10; severe learning disabilities; and insufficient understanding of English to complete assessments. A population-based and demographically representative sample of controls resident in the same catchment areas as cases, aged 18–64 years, and without a current or past history of psychotic disorder was recruited using a mixture of quota and random sampling. First, quotas were set, based on the 2011 Census of the local population, for sex, age group, and ethnic group, in order to: 1) ensure recruitment of a sample of controls that reflected the demographic profile, and 2) ensure we had a sufficient number of controls from black Caribbean and black African groups for analyses by ethnic group. Second, two sampling frames were used to fill these quotas: a) general practitioner (GP) lists and 2) the postal address file (PAF). These methods are described in more detail elsewhere (Beards et al., in press). All potential controls were screened for current or past history of psychosis using the Psychosis Screening Questionnaire (Bebbington and Nayani, 1995).

### 2.2 Measures

Sections of the Childhood Experience of Care and Abuse (CECA) schedule (Bifulco et al., 1994), an in-depth face-to-face semi-structured interview, were used to collect data on physical, sexual and emotional abuse perpetrated by a carer/guardian, or another relative or individual at least five years older than the recipient. Severity was rated on a 4-point scale: none, some, moderate, and marked and dichotomised in accordance with the CECA manual into none/mild versus moderate/marked. The CECA has previously been shown to have a high degree of inter-rater reliability (Bifulco et al., 1994) and reasonable levels of validity (Bifulco et al., 1997). In order to reduce the likelihood of observer bias, and to increase accuracy and consistency for all interviews, all researchers who administered the CECA interview underwent intensive training, and all CECA ratings were subsequently made by consensus within the research team, using detailed notes taken during the interviews. The Childhood Trauma Questionnaire (CTQ) (Bernstein and Fink, 1998; Bernstein et al., 2003) is a self-report instrument consisting of 28 items measuring physical, sexual, and emotional abuse, physical and emotional neglect. Each subscale contains five items, rated on a five-point Likert-type scale from 1 never to 5 very often true, equating to scores in the range of 5–25 for each subscale. Established cut-offs for the CTQ were used to create dichotomous variables indicating none/mild forms versus moderate/severe forms of each type of abuse (Bernstein et al., 2003). For comparability we focus here just on the abuse subscales.

### 2.3 Ethics

Ethical approval for was obtained from the South London and Maudsley NHS Foundation Trust and the Institute of Psychiatry Research Ethics Committee (Ref: 321/05, including amendments 1 to 9).

### 2.4 Statistical analysis

Acknowledging the limitations of both self-report and interviewer-based measures of childhood abuse, and that there is no gold standard measure, we examined the level of agreement between the two methods (Reitsma et al., 2009) using the CECA ratings as our reference measure and compared these with CTQ ratings.

Areas under the curve (AUCs), determined from receiver operating characteristic (ROC) analyses, were used to quantify the accuracy of the dichotomised CTQ subscales against the CECA ratings in discriminating individuals with and without moderate to severe levels of abuse. We used the following guideline for evaluating AUC values: <0.70, poor; 0.70 to 0.79, fair; 0.80 to 0.89, good; and 0.90 to 1.00, excellent (Swets, 1988). Sensitivity and specificity analyses were conducted to determine the proportion of cases with psychosis and controls, independently, who reported positive or negative histories of abuse on the CTQ and identified as such on the CECA. Kappa coefficients were also calculated to determine the overall strength of agreement between these two measures and we used the following guidelines for evaluating them: <0.10, virtually none; 0.11 to 0.40, slight; 0.41 to 0.60, fair; 0.61 to 0.80, moderate; and 0.81 to 1.00, substantial (Shrout, 1998). Convergent validity (i.e. the degree to which two tests designed to assess the same construct are related) between the CECA and CTQ ratings was examined using tetrachoric correlations, which take into account the underlying distribution of the binary estimations from continuous variables.

## 3 Results

Data were available on 234 cases and 293 controls who had completed both the CECA and CTQ. Compared with controls, a greater proportion of the cases were men (64% vs 52%), were less likely to consider themselves as white British (27% vs. 43%), and were younger (cases median age: 26, interquartile range [IQR] 22–32; controls median age: 32, IQR 26–43).

**Fig. 1:**
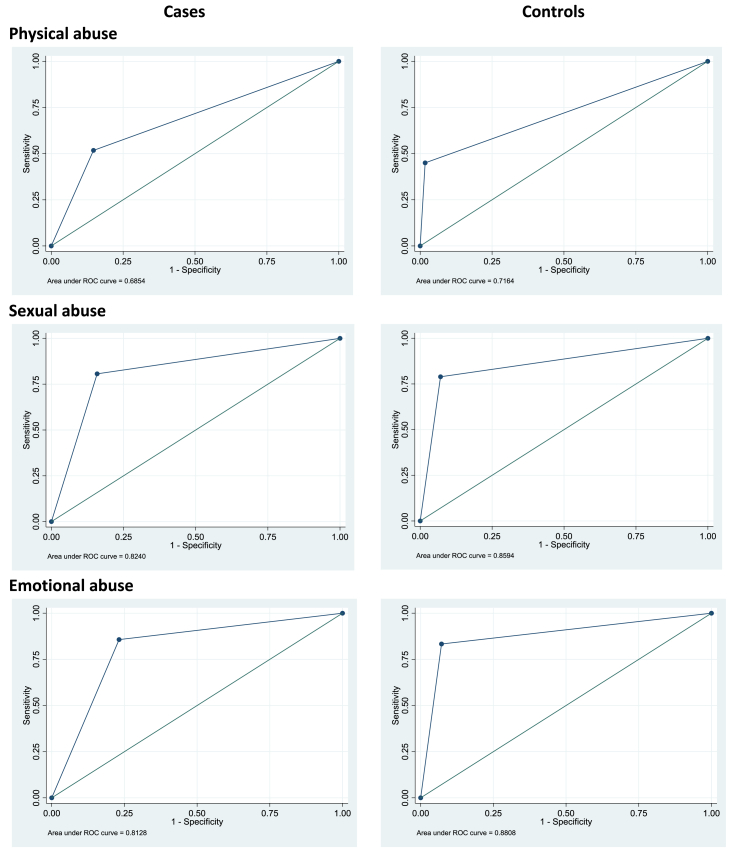
Receiver Operating Characteristic curves of the proportion of individuals who reported positive or negative histories of physical, sexual, and emotional abuse for cases and controls rated by the Childhood Trauma Questionnaire (CTQ) compared to the ratings made by interviewers on the Childhood Experience of Care and Abuse (CECA) interview (i.e. sensitivity and specificity). Established cut-offs for the CTQ were employed to represent instances of none to mild versus moderate to severe levels of abuse. The diagonal line which runs from the lower left corner to the upper right corner reflects the characteristics of a scale performing no better than chance. The better the discriminating ability of the scale, the closer the curve will approach the upper left corner.

The ROC curves for each type of abuse, separately for cases and controls, are shown in Fig. 1. Overall, in the sample as a whole, compared with the CECA interview, the CTQ resulted in around twice as many reports of sexual (18% versus 10%) and emotional (20% versus 9%) abuse. The reverse was observed for physical abuse (19% when measured by the CTQ, 28% for the CECA interview). When the CECA ratings of abuse were used as the criterion, the area under the ROC curve revealed that the CTQ had varying levels of discriminating ability for different forms of abuse: i.e., poor for physical abuse among cases (AUC 0.69), fair for physical abuse among controls (AUC 0.72), and good for sexual abuse and emotional abuse among cases and controls (all AUCs between 0.81 and 0.88).

Overall levels of agreement (kappa) and sensitivity and specificity are shown in Table 1. Levels of agreement between CTQ and CECA ratings were similar in cases and controls for both sexual and emotional abuse. These kappa coefficients represent fair levels of agreement (Shrout, 1998). For physical abuse, levels of agreement between self-report and interview ratings were higher in controls than cases, the latter falling just short of fair agreement between the two measures.

**Table 1:** Level of agreement, sensitivity and specificity of CECA and CTQ childhood abuse ratings.

	Childhood Experience of Care and Abuse (CECA) interview (reference)^a^																
	Cases									Controls							
	Absence n (%)		Presence n (%)			Sensitivity (95% CI)	Specificity (95% CI)	Kappa (95% CI)	P	Absence n (%)		Presence n (%)		Sensitivity (95% CI)	Specificity (95% CI)	Kappa (95% CI)	P
**Childhood Trauma Questionnaire (CTQ)**																	
Physical abuse^b^																	
Presence	21	(14.7)	44		(51.8)	51.8 (45.3–58.3)	85.3 (80.7–89.9)	0.39 (0.27–0.51)	<0.001	4	(1.7)	27	(45.0)	45.0 (39.3–50.7)	98.3 (96.8–99.8)	0.53 (0.40–0.66)	<0.001
Absence	122	(85.3)	41		(48.8)					228	(98.3)	33	(55.0)				
Sexual abuse^c^																	
Presence	29	(15.8)		25	(80.7)	80.7 (75.4–85.9)	84.2 (79.3–89.1)	0.50 (0.36–0.64)	<0.001	19	(7.1)	15	(79.0)	79.0 (74.2–83.7)	92.9 (90.0–95.9)	0.53 (0.36–0.69)	<0.001
Absence	154	(84.2)		6	(19.3)					250	(92.9)	4	(21.0)				
Emotional abuse^d^																	
Presence	44	(23.2)	30		(85.7)	85.7 (81.1–90.3)	76.8 (71.3–82.4)	0.43 (0.31–0.55)	<0.001	20	(7.2)	10	(83.3)	83.3 (79.1–87.6)	92.8 (89.9–95.8)	0.44 (0.26–0.63)	<0.001
Absence	146	(76.8)	5		(14.3)					259	(92.8)	2	(16.7)				

a Absence of abuse defined as a CECA rating of none/mild; Presence of abuse defined as a CECA rating of moderate/severe

b Absence of physical abuse defined as CTQ total scores of 5–9; Presence defined as CTQ total scores of 10+

c Absence of sexual abuse defined as CTQ total scores of 5–7; Presence defined as CTQ total scores of 8+

d Absence of emotional abuse defined as CTQ total scores of 5–12; Presence defined as CTQ total scores of 13+

Sensitivity and specificity were fair to high in both cases and controls, with the proportion of individuals who reported abuse on the CTQ and identified as such on the CECA (i.e. sensitivity) highest for emotional abuse. The proportion of individuals who did not report abuse on the CTQ and were identified as such on the CECA (i.e. specificity) was highest for physical abuse in both groups (Table 1).

Finally, we found good convergent validity between CECA and CTQ ratings of abuse in cases and controls for both sexual abuse and emotional abuse (Table 2). However, for reports of physical abuse, convergent validity was stronger in controls than cases.

**Table 2:** Convergent validity of CECA and CTQ childhood adversity ratings.

	Cases			Controls		
	r_t_	s.e.	P	r_t_	s.e.	P
Physical abuse‡	0.61	0.08	<0.001	0.87	0.05	<0.001
Sexual abuse^a^	0.80	0.07	<0.001	0.86	0.06	<0.001
Emotional abuse^b^	0.78	0.07	<0.001	0.86	0.07	<0.001

CECA, Childhood Experience of Care and Abuse interview. CTQ, Childhood Trauma Questionnaire. † Absence of abuse defined as a CECA rating of none/mild; Presence of abuse defined as a CECA rating of moderate/severe; ‡ Absence of physical abuse defined as CTQ total scores of 5-9; Presence defined as CTQ total scores of 10+

a Absence of sexual abuse defined as CTQ total scores of 5–7; Presence defined as CTQ total scores of 8+.

b Absence of emotional abuse defined as CTQ total scores of 5–12; Presence defined as CTQ total scores of 13+.

## 4 Discussion

In this case-control study, we found that accuracy, strength of agreement, and convergent validity were broadly similar in cases with first-episode psychosis and population-based controls for two distinct retrospective measures of childhood abuse, a brief questionnaire and a more comprehensive interview. Specifically, findings on sexual and emotional abuse showed overall moderate to high proportion of individuals who reported positive or negative histories of abuse on the CTQ compared to the CECA, reasonably high tetrachoric correlations between the measures (i.e. high convergent validity), and fair levels of agreement of CECA and CTQ ratings in both groups. However, for physical abuse, the proportion of individuals who did not report abuse on the CTQ, but were identified as having been physically abused on the CECA was rather high in cases and controls. Also, convergent validity of self-report and interviewer ratings of physical abuse were marginally stronger in controls. In short, reports of abuse established from these two measures were broadly comparable, and there was no strong evidence that accuracy of abuse established from a self-report questionnaire was lower in cases compared with controls.

These findings should be viewed in light of several potential methodological limitations. As for many if not most measures in mental health research, there is no gold standard instrument for childhood abuse, and in the absence of any objective indicators, general bias in retrospective reports due to forgetting, repression of traumatic events, and embarrassment (Susser and Widom, 2012) cannot be ruled out. Nevertheless, we found no differences in the convergent validity of sexual and emotional abuse reports in cases and controls, and only marginal differences concerning physical abuse. Therefore, the results do not suggest that reports of abuse by cases are less valid than reports by controls, thereby allaying some of the concerns about the potential influence of recall bias on reported associations between abuse and psychosis (Lysaker et al., 2005; Saykin et al., 1991; Susser and Widom, 2012; Young et al., 2001). In addition, the classification systems employed here, for example, for the kappa coefficients to assess strength of agreement between measures, are ultimately fairly arbitrary; the kappa coefficients of 0.43–0.53 as reported in this study do reflect only limited agreement between the CECA and CTQ.

In contrast to previous claims that relying on retrospective reports of childhood abuse may affect the validity of findings on the reported associations between childhood adversity and psychosis (Matheson et al., 2013; Morgan and Gayer-Anderson, 2016; Susser and Widom, 2012; Varese et al., 2012), our data broadly support the validity of this method of data collection in this context. Consistent with the findings by Fisher et al. (2011), we found good convergent validity of patients’ retrospective reports. Reports of childhood abuse by people with mental disorder have been shown to be reasonably reliable over time (Fisher et al., 2011; Goodman et al., 1999; Herman and Schatzow, 1987). In line with this, the meta-analysis by Varese et al. (2012) reported similar findings for the association between childhood adversity and psychosis in cross-sectional and in prospective and quasi-prospective studies. Prospective longitudinal studies, where abuse is measured prior to the onset of psychotic disorder, would of course be the ideal design. However, they equally come with their own challenges; prospective studies are i) prohibitively expensive due to the time-lag between exposure and disorder onset, and the large numbers of individuals that would need to be followed up given the rarity of the disorder; and ii) it would be very difficult to collect such material contemporaneously, especially regarding abuse within the family; parental informants might intentionally or unknowingly provide inaccurate accounts, there are obviously ethical and practical issues associated with interviewing the children themselves, and administrative reports of abuse capture only a minority of those exposed. Given these points, our findings broadly support the use of these retrospective measures as a valid and economic alternative in case-control studies.

In conclusion, the findings reported in this paper provide no strong evidence that the validity of reports of abusive experiences in childhood systematically differs across patients with psychosis and controls. Both of these measures may be used in future research, including case-control studies, of the complex relationship of childhood abuse and psychosis, though time-permitting, the CECA interview may be preferable as it provides more detailed contextual information about the abuse experiences (e.g. the perpetrator, severity, and frequency of abuse).

## CRediT authorship contribution statement

**Charlotte Gayer-Anderson:** Investigation, Data curation, Formal analysis, Writing - original draft. **Ulrich Reininghaus:** Formal analysis, Writing - review & editing. **Isabell Paetzold:** Writing - original draft. **Kathryn Hubbard:** Investigation, Data curation, Formal analysis. **Stephanie Beards:** Investigation, Data curation. **Valeria Mondelli:** Conceptualization, Writing - review & editing. **Marta Di Forti:** Conceptualization, Writing - review & editing. **Robin M. Murray:** Conceptualization, Writing - review & editing. **Carmine M. Pariante:** Conceptualization, Writing - review & editing. **Paola Dazzan:** Conceptualization, Writing - review & editing. **Thomas J. Craig:** Conceptualization, Writing - review & editing. **Helen L. Fisher:** Conceptualization, Writing - review & editing. **Craig Morgan:** Conceptualization, Funding acquisition, Writing - review & editing.

## Declarations of competing interest

None.

## Appendix A Supplementary data

Data Profile.xml Data Profile.xml

## References

[bib1] Beards, S., Fisher, H.L., Gayer-Anderson, C., Hubbard, K., Reininghaus, U., Craig, T.J., Di Forti, M., Mondelli, V., Pariante, C., Dazzan, P., Murray, R.M., Morgan, C., (in press). Threatening and intrusive life events and difficulties and psychotic disorder. Schizophr. Bull. 10.1093/schbul/sbaa005 PMC7342097 32047940

[bib2] Bebbington P., Nayani T. (1995). The psychosis screening questionnaire. Int. J. Methods Psychiatr. Res..

[bib3] Bernstein D.P., Fink L. (1998). Childhood Trauma Questionnaire: A Retrospective Self-Report.

[bib4] Bernstein D.P., Stein J.A., Newcomb M.D., Walker E., Pogge D., Ahluvalia T., Stokes J., Handelsman L., Medrano M., Desmmond D., Zule W. (2003). Development and validation of a brief screening version of the Childhood Trauma Questionnaire. Child Abuse Negl..

[bib5] Bifulco A., Brown G.W., Harris T.O. (1994). Childhood experiences of Care and abuse (CECA): a retrospective interview measure. JCPP (J. Child Psychol. Psychiatry).

[bib6] Bifulco A., Brown G.W., Lillie A., Jarvis J. (1997). Memories of childhood neglect and abuse: corroboration in a series of sisters. JCPP (J. Child Psychol. Psychiatry).

[bib7] Colman I., Kingsbury M., Garad Y., Zeng Y., Naicker K., Patten S., Jones P.B., Wild T.C., Thompson A.H. (2016). Consistency in adult reporting of adverse childhood experiences. Psychol. Med..

[bib8] Dill D.L., Chu J.A., Grob M.C., Eisen S.V. (1991). The reliability of abuse history reports: a comparison of two inquiry formats. Compr. Psychiatr..

[bib9] Fisher H.L., Craig T.K., Fearon P., Morgan K., Dazzan P., Lappin J., Hutchinson G., Doody G.A., Jones P.B., McGuffin P., Murray R.M., Leff J., Morgan C. (2011). Reliability and comparability of psychosis patients' retrospective reports of childhood abuse. Schizophr. Bull..

[bib10] Goodman L.A., Thompson K.M., Weinfurt K., Corl S., Acker P., Mueser K.T., Rosenberg S.D. (1999). Reliability of reports of violent victimization and posttraumatic stress disorder among men and women with serious mental illness. J. Trauma Stress.

[bib11] Herman J.L., Schatzow E. (1987). Recovery and verification of memories of childhood sexual trauma. Psychoanal. Psychol..

[bib12] Lysaker P.H., Beattie N.L., Strasburger A.M., Davis L.W. (2005). Reported history of child sexual abuse in schizophrenia. J. Nerv. Ment. Dis..

[bib13] Matheson S.L., Shepherd A.M., Pinchbeck R.M., Laurens K.R., Carr V.J. (2013). Childhood adversity in schizophrenia: a systematic meta-analysis. Psychol. Med..

[bib14] Morgan C., Fisher H. (2007). Environment and schizophrenia: environmental factors in schizophrenia: childhood trauma--a critical review. Schizophr. Bull..

[bib15] Morgan C., Gayer-Anderson C. (2016). Childhood adversities and psychosis: evidence, challenges, implications. World Psychiatr..

[bib16] Read J., van Os J., Morrison A.P., Ross C.A. (2005). Childhood trauma, psychosis and schizophrenia: a literature review with theoretical and clinical implications. Acta Psychiatr. Scand..

[bib17] Reitsma J.M., Rutjes A.W.S., Khan K.S., Coomarasamy A., Bossuyn P.M. (2009). A review of solutions for diagnostic accuracy studies with an imperfect or missing reference standard. J. Clin. Epidemiol..

[bib18] Roy C.A., Perry J.C. (2004). Instruments for the assessment of childhood trauma in adults. J. Nerv. Ment. Dis..

[bib19] Saykin A.J., Gur R.C., Gur R.E., Mozley P.D., Mozley L.H., Resnick S.M., Kester D.B., Stafiniak P. (1991). Neuropsychological function in schizophrenia. Selective impairment in memory and learning. Arch. Gen. Psychiatr..

[bib20] Shrout P.E. (1998). Measurement reliability and agreement in psychiatry. Stat. Methods Med. Res..

[bib21] Susser E., Widom C.S. (2012). Still searching for lost truths about the bitter sorrows of childhood. Schizophr. Bull..

[bib22] Swets J.A. (1988). Measuring the accuracy of diagnostic systems. Science.

[bib23] Varese F., Smeets F., Drukker M., Lieverse R., Lataster T., Viechtbauer W., Read J., van Os J., Bentall R.P. (2012). Childhood adversities increase the risk of psychosis: a meta-analysis of patient-control, prospective- and cross-sectional cohort studies. Schizophr. Bull..

[bib24] Young M., Read J., Barker-Collo S., Harrison R. (2001). Evaluating and overcoming barriers to taking abuse histories. Prof. Psychol. Res. Pract..

